# Progress, clinical application and challenges of non-invasive prenatal testing for monogenic diseases

**DOI:** 10.3389/fped.2026.1734842

**Published:** 2026-02-04

**Authors:** Chengcheng Wang, Dongxia Hou, Gang Wang, Xi Wu, Zhiyong Zhou, Lina Yang, Ying Liu, Xiaohua Wang

**Affiliations:** 1 Inner Mongolia Medical University, Hohhot, China; 2 Department of Genetics and Eugenics, Inner Mongolia Autonomous Region Maternal and Child Health Hospital, Hohhot, China; 3 Inner Mongolia Autonomous Region Engineering Research Center of Medical Genetics, Hohhot, China; 4 Xing ‘an League People’s Hospital, Wulanhot, China

**Keywords:** analytical strategies, challenges, clinical application, monogenic diseases, non-invasive prenatal, sequencing technology

## Abstract

Monogenic diseases represent a significant healthcare challenge, characterized by their heritable nature and substantial disease burden. While non-invasive prenatal testing (NIPT) is well-established for aneuploidy, its application has rapidly expanded to monogenic conditions. However, current monogenic NIPT faces challenges including low sensitivity for maternally inherited variants, limited fetal DNA fraction, high cost, lack of standardized clinical validation, and complex ethical and counseling considerations. This review systematically summarizes the major technological approaches, current clinical applications, and core challenges of NIPT for monogenic diseases. It further discusses the underlying scientific issues and translational barriers associated with existing technical limitations, and offers perspectives on future directions. The aim is to provide a reference framework for advancing research and promoting standardized clinical implementation in this field.

## 1 Introduction

Monogenic diseases, stemming from single-gene pathogenic variants, are a significant cause of congenital morbidity and mortality. These conditions disproportionately affect infants and young children, often resulting in chronic disability or premature death, with therapeutic options frequently limited. More than 7,000 such monogenic diseases (1) are cataloged in the OMIM database (2). They follow diverse inheritance patterns—autosomal dominant, autosomal recessive, and X-linked. *De novo* mutations are a significant contributor to dominant diseases (70%–80% of cases) but are less common in recessive disorders (<10%) (3, 4), complicating prevention, as parents often lack prior risk indicators. While preconception carrier screening is effective for inherited conditions, it's limited for *de novo* mutations. Thus, accurate prenatal diagnosis of fetal monogenic diseases remains a pressing and unresolved challenge for preventing congenital disabilities and guiding perinatal care.

Conventional prenatal screening relies on maternal serum assays and ultrasound to evaluate fetal risk. These methods are established for assessing aneuploidy, neural tube defects, and structural anomalies. However, these modalities have limited sensitivity and specificity for monogenic diseases. As a result, traditional screening approaches are insufficient for comprehensive detection of monogenic diseases, underscoring the critical need for molecular diagnostic strategies in prenatal care. The 1997 identification of cell-free fetal DNA (cffDNA) in maternal plasma marked a watershed in prenatal diagnostics, enabling non-invasive access to fetal genetic material early in pregnancy (5). Early cffDNA-based non-invasive prenatal testing (NIPT) primarily focused on chromosomal aneuploidies, yielding markedly improved detection rates compared to traditional serum screening. However, extending NIPT to monogenic diseases was initially constrained by low sensitivity for detecting point mutations and small indels amid the high maternal DNA background. Recent advances in high-throughput sequencing, molecular enrichment, and computational analysis have substantially broadened NIPT capabilities, facilitating more robust non-invasive detection of monogenic diseases.

**Figure 1:**
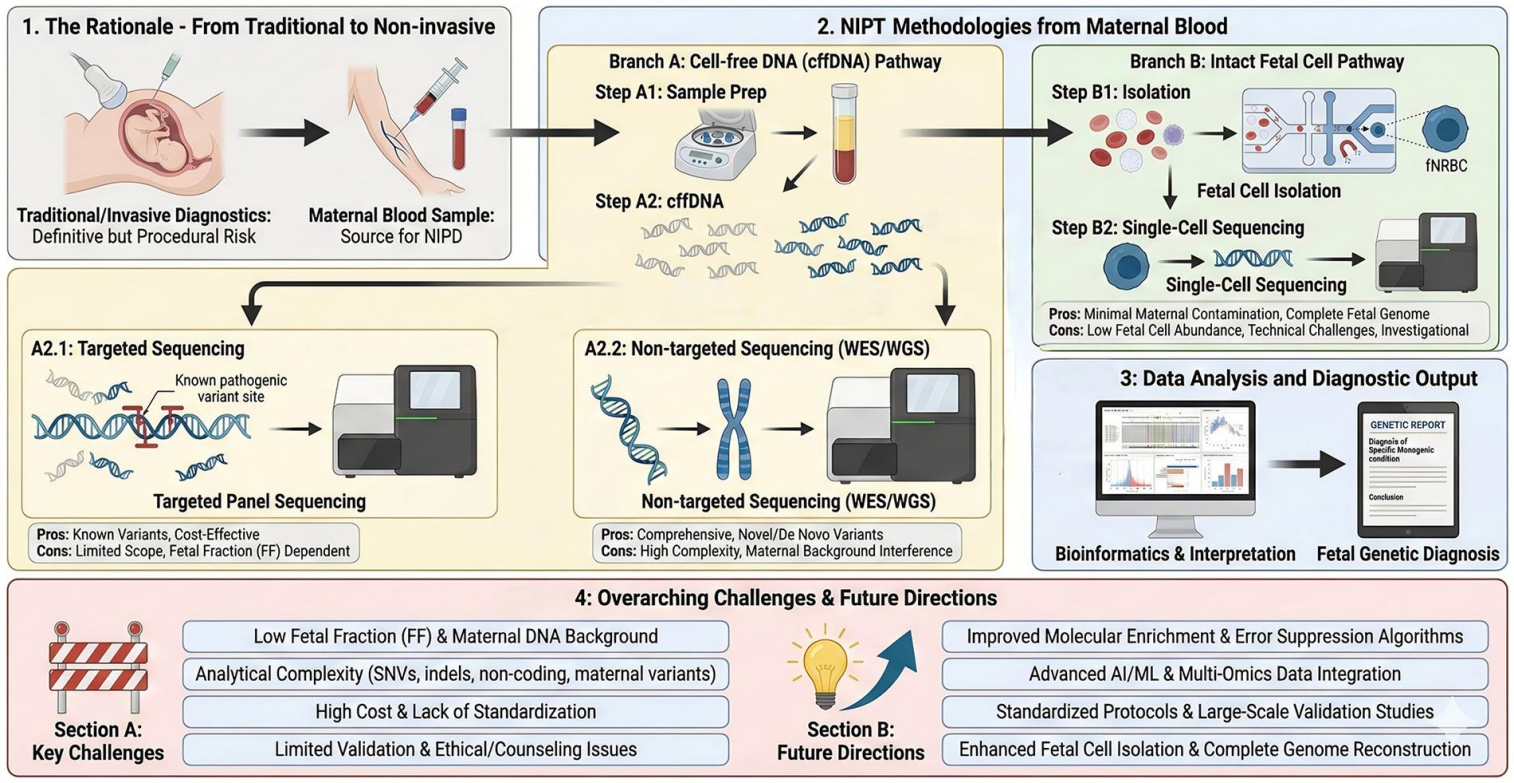
Overview of non-invasive testing of fetal monogenic diseases. This figure systematically outlines the core technical pathways of NIPT, transitioning from traditional invasive diagnostics to maternal blood-based approaches. It details two main methodological branches: the cell-free fetal DNA (cffDNA) pathway (with targeted and whole-exome/genome sequencing sub-branches) and the intact fetal cell pathway (involving isolation and single-cell sequencing). Each branch includes key procedural steps and summarizes respective advantages and limitations. The subsequent stage of bioinformatics analysis leading to a fetal genetic diagnosis is shown. Finally, the diagram highlights overarching current challenges (e.g., low fetal fraction, analytical complexity, cost) and future research directions (e.g., improved enrichment techniques, AI/ML integration, standardization). This structured overview provides a comprehensive framework for understanding the technological landscape and evolution of NIPT.

NIPT approaches for monogenic diseases include targeted, non-targeted (genome-wide), and single-cell sequencing. Targeted assays, like multigene panels, efficiently detect known pathogenic variants. Non-targeted strategies, such as fetal whole-exome/genome sequencing, expand the diagnostic scope to detect *de novo* variants and a wider range of variant classes (6). Recently, isolating circulating fetal cells for single-cell genomic sequencing has enabled more complete fetal genome reconstruction, addressing limitations of cffDNA and enhancing comprehensive prenatal diagnosis (7). Concurrent advancements in molecular enrichment, error suppression, and analytical pipelines continue to refine NIPT sensitivity and specificity, actively shaping its clinical implementation. Accurate analytical frameworks are crucial for monogenic NIPT, employing strategies such as relative mutation dosage (RMD) and relative haplotype dosage (RHDO) to infer fetal genotype from cffDNA (8). Despite progress, sensitivity and specificity remain suboptimal for some variants and clinical contexts. Key practical limitations include low fetal fraction (FF) in maternal plasma, technical/bioinformatics errors, high per-sample costs, ethical/counseling complexities, and a lack of standardized procedures (9). Addressing these constraints—through improved molecular enrichment, error-suppression algorithms, validated multiethnic data, and consensus guidelines—is vital for integrating NIPT for monogenic diseases into routine clinical practice. This review systematically appraises the current state of NIPT for monogenic diseases, focusing on recent technological advances, clinical applicability, and implementation challenges. It identifies principal bottlenecks, such as analytical limitations, validation gaps, and ethical/cost considerations. The review proposes priority directions to facilitate robust clinical integration and broaden NIPT's utility in prenatal care (Figure 1).

## 2 NIPT techniques for monogenic diseases

### 2.1 Targeted capture sequencing for NIPT

In 2000, Saito et al. (10) provided early proof-of-principle that a fetal monogenic disorder—achondroplasia, an autosomal dominant condition—could be detected in maternal plasma. The investigators designed primers specific to the FGFR3 c.1138G > A substitution to amplify fetal mutant alleles selectively. By confirming that paternal polymorphisms absent from the maternal genome were present in the amplified DNA, they verified the fetal origin of the amplified DNA. They excluded maternal-derived interference, demonstrating that the mutation arose from the fetus. This study represented a seminal advance in targeted capture and allele-specific detection for NIPT.

Early non-invasive molecular assays for fetal monogenic diseases relied on allele-specific PCR and restriction fragment analysis but have since transitioned to next-generation sequencing (NGS)—based workflows. NGS enables expansion from single-disease assays to multi-gene panels targeting high-prevalence autosomal-dominant conditions (11, 12). Multicenter prospective studies have demonstrated exceptional performance for optimized targeted enrichment strategies: for example, coordinated allele-aware targeted enrichment sequencing (COATE-seq), which integrates unique molecular identifiers (UMIs) with multidimensional bioinformatic algorithms to suppress enrichment bias and sequencing error, reported 100% sensitivity and specificity across a panel of 92 monogenic diseases (13). Independent evaluations of UMI-based approaches and rigorous clinical validation cohorts have similarly documented near-perfect detection of single-nucleotide variants, particularly for paternally inherited and *de novo* mutations (11, 14–17). Targeted NGS has also shown utility in preimplantation genetic testing and prenatal management for specific conditions, including monogenic kidney diseases and Fanconi anemia (18–20). Despite these advances, challenges remain in resolving low-frequency maternal origin alleles, ensuring broad population validation, and establishing standardized clinical pipelines.

Targeted sequencing has substantially advanced NIPT of monogenic diseases and remains comparatively cost-effective; however, essential limitations persist. Its performance is highly dependent on fetal fraction (FF), with low FF compromising sensitivity and specificity (9). Mitigation strategies include increasing sequencing depth according to gestational age and orthogonal confirmation using droplet digital PCR (ddPCR) (21). Expanding targeted panels to cover a wider set of phenotype-relevant genes, or adopting broader approaches such as whole-exome (WES) or whole-genome sequencing (WGS), can improve diagnostic yield for complex or phenotypically ambiguous cases and reduce missed diagnoses (22, 23).

Most reported NIPT studies have enrolled high-risk pregnancies, limiting generalizability to the unselected obstetric population. Lower pretest probability in routine cohorts reduces the positive predictive value and may increase false-positive and false-negative rates (13). To establish real-world performance, large prospective, population-based cohorts are needed to derive representative sensitivity, specificity, and predictive values and to refine analytical thresholds and algorithms. Integrating genetic results with clinical variables—family history, maternal age, and ultrasound findings—will improve risk stratification, reduce overreliance on molecular data, and enhance diagnostic robustness across diverse clinical settings (Table 1).

**Table 1:** Development of NIPT technology for monogenic diseases.

Time	Country	Monogenic Disease Category	Application Technology	Related Gene(s)	Ref
2000	Japan	Achondroplasia	Targeted PCR	*FGFR3*	(10)
2012	UK	Achondroplasia, Thanatophoric Dysplasia	PCR-RED	*FGFR3; COL2A1, FGFR1, FGFR2, FGFR3*	(24)
2013	UK	Apert Syndrome, Cystic Fibrosis, FGFR2-related Craniosynostosis, Crouzon Syndrome with Acanthosis Nigricans	NGS	*FGFR2; CFTR*	(25)
2015	NL	Huntington	ddPCR	*HTT*	(26)
2018	China	Achondroplasia	NGS	*FGFR3*	(27)
2019	US	Achondroplasia, Pfeiffer Syndrome, etc. (27 types of fetal *de novo* dominant monogenic diseases)	Target Region Capture + UMI Library Construction with High-Throughput Sequencing	*FGFR3; FGFR2; TSC1, TSC2*, etc.	(28)
2023	China	92 types of monogenic dominant genetic disorders caused by 64 genes	COATE-seq (Collaborative Allele-specific Target Enrichment Sequencing)	*FGFR3, PTPN11*, and other 64 genes	(13)

### 2.2 Non-targeted sequencing for NIPT

Targeted NIPT assays are typically limited to established pathogenic loci and, therefore, may miss *de novo* or otherwise pathogenic variants outside predefined regions. Non-invasive fetal whole exome sequencing (WES) and whole genome sequencing (WGS) overcome this constraint by providing an unbiased, genome-wide survey that enables detection of *de novo* mutations and novel variant classes across coding and noncoding regions. Compared with targeted panels, WES/WGS increases the likelihood of identifying unexpected or previously uncharacterized pathogenic variants, offering a more comprehensive and potentially earlier means of fetal genetic diagnosis—albeit with greater analytical complexity, higher sequencing demand, and increased interpretive challenges.

#### 2.2.1 Whole exome sequencing

Approximately 80% of monogenic diseases are attributable to variants within exonic regions, making non-invasive assessment of the fetal exome an attractive strategy for early prenatal detection. Non-invasive whole exome sequencing (WES) employs hybrid capture of coding regions—roughly 2% of the genome—combined with parental or familial genotypes to infer fetal status. Recent studies have demonstrated the practical potential and limitations of this approach. Brand et al. (22) applied capture sequencing across ∼22,995 genes in cffDNA from 51 pregnancies, achieving a median sensitivity of 96.3% for *de novo* and paternally inherited single nucleotide variants (SNVs) and 100% detection for certain structural events, but lower performance for insertions/deletions (median sensitivity ∼81.9%) and substantially reduced sensitivity for maternally inherited heterozygous variants. Low fetal fraction and inadequate sequencing depth were identified as principal determinants of reduced accuracy (22, 29). Ultra-deep exome sequencing of cffDNA (mean coverage ∼4,548×) has achieved complete detection of pathogenic *de novo* variants in small cohorts (30). Yet, the prohibitive cost of such depth currently limits clinical scalability. Together, these data indicate that exome-focused NIPT can substantially broaden diagnostic reach but requires optimized fetal fraction management, error-suppression methods, and cost-effective sequencing strategies to enable broader implementation.

Non-invasive whole-exome sequencing (WES) holds promise for prenatal screening for monogenic diseases but has inherent technical and interpretive constraints distinct from those of invasive diagnostic WES. Non-invasive WES analyzes cell-free DNA in maternal plasma, a mixture of low-fraction, fragmented fetal DNA and abundant maternal DNA; this composition necessitates ultra-deep sequencing and parental genotypes for bioinformatics deconvolution, and it limits sensitivity for maternal heterozygous variants and indels. As a result, non-invasive WES functions as an advanced screening modality whose findings are probabilistic rather than definitive. By contrast, invasive WES—performed on fetal cells obtained via amniocentesis or chorionic villus sampling—provides uncontaminated fetal DNA, enabling high-confidence detection of virtually all variant classes at standard sequencing depths and is therefore regarded as the diagnostic gold standard (31), albeit with a small risk of procedural miscarriage. Thus, non-invasive and invasive WES are complementary: the former offers a safer, lower risk option for broad screening, while the latter delivers definitive diagnoses when clinical certainty is required (32).

#### 2.2.2 Whole genome sequencing

Non-invasive whole genome sequencing (WGS) extends prenatal genetic screening beyond the exome by interrogating both coding and noncoding regions implicated in gene regulation and chromosomal integrity—including promoters, enhancers, telomeres, and centromeres—thereby enabling a more comprehensive, genome-level assessment of potential pathogenic variation. Clinical application of non-invasive WGS has identified pathogenic KMT2D variants in fetuses with Kabuki syndrome that were not detected by invasive exome sequencing in the same cohort, illustrating WGS's capacity to reveal diagnoses missed by exome targeted assays (33). However, WGS introduces substantial interpretive complexity, particularly for noncoding variants. In addition to ACMG criteria, computational annotation frameworks—such as DeepSEA, GWAVA, and CADD—are routinely applied to prioritize noncoding and regulatory variants. Still, these predictions require careful contextualization and experimental validation (34, 35). A persistent technical limitation for non-invasive approaches remains accurate resolution of maternally inherited variants: reported accuracy for maternal SNV detection has been modest (approximately 54%–64% in several studies), motivating development of methods such as ultra deep sequencing, relative allele dosage analyses, and specialized software pipelines (e.g., Hoobari) to improve maternal allele discrimination and overall diagnostic sensitivity (36). Continued methodological refinement and rigorous validation are therefore required before non-invasive WGS can be routinely applied for comprehensive prenatal monogenic diagnosis.

Advances in analytical methods have improved the detection of maternally inherited single-nucleotide variants (SNVs) from maternal plasma. Integrative approaches that combine fragmentomics, statistical modeling, and machine learning now achieve mean area under the receiver operator characteristic curve (AUC) values of ∼0.81 for maternal SNV prediction, with AUCs up to 0.86 for homozygous biallelic variants (23). Performance is further enhanced within coding regions of clinically relevant genes, where higher mappability, reduced repetitive sequence content, and lower reference bias yield superior discrimination metrics (23, 36). These improvements markedly increase sensitivity compared with earlier methods, although challenges remain for comprehensive, routine clinical implementation.

Accurate genotyping of parental compound heterozygous variants remains a significant obstacle for NIPT. Inspired by the success of deep learning in biology, Schwammenthal et al. extended the Hoobari framework to develop deepHoobari, a neural network–based method for predicting deleterious parental compound-heterozygous genotypes from non-invasive whole-genome data (37). The model ingests multidimensional features—sequence context, fragment length distributions, and methylation signatures—and employs ReLU activations within hidden layers trained on tens of thousands of labeled examples. In a validation set of 100 samples with known parental compound heterozygosity, deepHoobari achieved high accuracy, demonstrating the potential of deep learning to resolve complex inheritance patterns in cell-free DNA and improving the feasibility of genome-wide non-invasive detection of compound heterozygous monogenic disease.

Despite advances in detecting maternally inherited SNVs and parental biallelic variants, the clinical sensitivity and validity of non-invasive assays remain suboptimal. Emerging methods—integrating fragmentomics, deep learning, ultra-deep sequencing, and allele-aware algorithms—have improved discrimination of maternal and fetal alleles and shown robust performance in limited cohorts, but require larger, standardized validation studies before routine clinical adoption. A fundamental limitation persists: cell-free fetal DNA in maternal plasma is highly fragmented and cannot by itself reconstruct a continuous fetal genome. To overcome this, current research is converging on a combination of strategies—single-molecule long-read sequencing, fetal-maternal cell-free DNA haplotyping, and integrative analysis with parental genomes—to increase genome completeness and diagnostic accuracy. Continued methodological refinement and large-scale prospective validation are essential to translate these approaches into reliable, comprehensive non-invasive prenatal testing.

### 2.3 Single-Cell sequencing

Single-cell sequencing is an emergent and rapidly advancing modality in prenatal genomics. Intact fetal cells circulating in maternal blood—including fetal nucleated red blood cells (fNRBCs), circulating trophoblast cells (CTCs), and fetal lymphocytes—harbor complete fetal genomes and transcriptomes, enabling simultaneous DNA and RNA profiling with minimal maternal background contamination compared with cell-free fetal DNA (cffDNA). Consequently, these cell types offer a direct route to high-confidence NIPT for monogenic diseases.

Recent studies have explored isolation and single-cell analysis of fNRBCs and CTCs for monogenic disease detection (38–40). For example, single-cell sequencing of fNRBCs has been applied to inherited hearing loss: fNRBCs were recovered in 8 of 9 families, and results concorded with invasive diagnostics in 88.9% of cases (41). Technical obstacles remain, principally the low abundance of fetal cells in maternal blood and the challenge of discriminating fNRBCs from rare maternal nucleated erythroid cells. Enrichment strategies—such as antibody-coated silica microspheres combined with multicolor immunofluorescence—have enabled the retrieval of hundreds of candidate fNRBCs from small blood volumes (42). Complementary approaches, including single-cell STR profiling of trophoblast-derived whole-genome products, have been used to confirm fetal cell origin before downstream analysis (43).

While single-cell NIPT promises near-complete fetal genomic resolution and reduced maternal interference, routine clinical translation requires improvements in capture efficiency, cell type specificity, and standardized workflows to ensure reproducibility and scalability.

Single-cell sequencing offers a transformative approach for non-invasive detection of monogenic diseases by enabling genomic and transcriptomic analysis at the level of individual fetal cells (41, 44, 45). Despite its considerable promise, the technology remains investigational and is not yet routinely deployable in clinical practice. Key technical limitations include variable and often low single-cell capture efficiency, challenges in unequivocally identifying and isolating true fetal cells, and resultant variability in diagnostic yield. These factors undermine reproducibility and constrain the method's current capacity to provide definitive clinical diagnoses, highlighting the need for improved enrichment techniques, standardized workflows, and large-scale validation before widespread clinical translation (Table 2).

**Table 2:** Comparison of characteristics of NIPT techniques for monogenic diseases.

Feature	Targeted Sequencing	Exome Sequencing (WES)	Whole Genome Sequencing (WGS)	Single-Cell Sequencing
Detection Scope	Known pathogenic mutations	All exonic variants	Coding + noncoding variants	Single-cell genomic/transcriptomic
Genetic Mode Coverage	Paternal: High *De novo*: Medium–High Maternal: Low	• Paternal/*de novo*: High • Maternal: Medium–Low	Paternal/*de novo*: High Maternal: Low	All modes: Theoretically feasible
Sensitivity	Paternal: >99% *De novo*: 88.9%–100% Maternal: <50%	*De novo*: 96.3% Maternal SNVs: ∼50%–60%	*De novo*: ∼96% Maternal: 54%–64%	*De novo*: Very Low Maternal: Low
Specificity	>99% (paternal/*de novo*)	∼98% (*de novo*) ∼90% (maternal)	∼95% (*de novo*) < 90% (maternal)	Not yet validated
Clinical Success Rate	100% (COATE-seq, 92 genes, *n* = 1000+)	96.3% (*de novo* SNVs, *n* = 51)	94.1% (PKU, RHDO, *n* = 61)	88.9% (hearing loss, *n* = 9)
Clinical Utility	High for known dominant disorders	Moderate for broad screening	Promising but interpretive burden high	Investigational
Limitations	Misses novel variants FF <4.5% reduces accuracy	Misses non-exonic variants Maternal SNVs hard to call	High cost Interpretation complex	Low yield No standard protocol
References	(6, 12, 27)	(22, 30–32)	(13, 29, 36)	(7, 31, 38, 41, 45)

## 3 Analytical strategies for NIPT

Accurate detection of fetal monogenic diseases requires not only high-resolution sequencing but also rigorous analytical frameworks to convert raw data into clinically actionable genotypes. In non-invasive prenatal testing, quantitative approaches such as relative mutation dosage (RMD) and relative haplotype dosage (RHDO) are widely employed to infer fetal genotype from cell-free DNA. RMD is well-suited for detecting paternally derived variants in dominant disorders and for identifying homozygous recessive states, whereas RHDO leverages parental haplotype phasing to resolve both dominant and recessive inheritance patterns by comparing relative allele or haplotype abundances (8, 9, 46). These methods, often combined with fetal fraction estimation, unique molecular identifiers, and error suppression algorithms, form the analytical backbone of current non-invasive monogenic disease workflows.

### 3.1 Relative mutant dose (RMD)

Relative mutation dosage (RMD) infers fetal genotype by precisely quantifying the proportional difference between wild-type and mutant alleles in maternal plasma cell-free DNA. Conceptually, non-pregnant heterozygous carriers exhibit a 1:1 allele ratio; if the fetus inherits a maternal heterozygous genotype, the ratio remains near equilibrium, whereas a fetal homozygous pathogenic genotype elevates the mutant allele fraction above 50% (and the converse holds for fetal wild type homozygosity). RMD relies on targeted probe design and high-throughput sequencing to achieve the necessary quantitative precision. The approach has been applied across multiple dominant and recessive conditions using multigene panels. Its performance at low fetal fraction has been improved through integration with highly quantitative technologies—digital PCR, droplet digital PCR (ddPCR), and circular single molecule amplification and resequencing (cSMART)—which enhance sensitivity and limit of detection in challenging samples.

**Figure 2:**
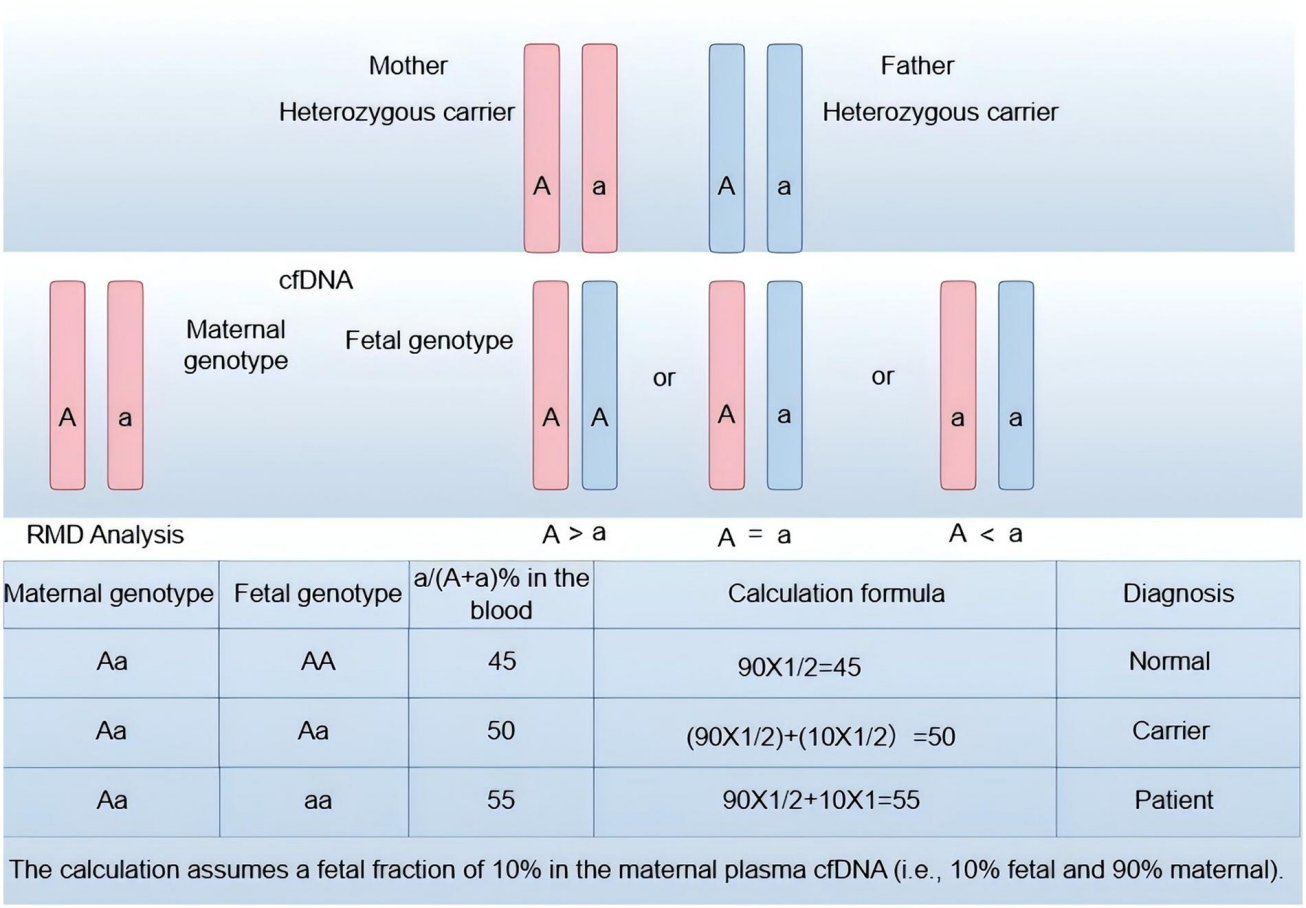
Principle of the relative mutation dose method. **(Top Panel)** Parental Genotypes: Both parents are heterozygous carriers (Genotype Aa) for the target mutation. The maternal alleles are depicted in pink, and the paternal alleles are depicted in blue. “A” represents the wildtype allele, and “a” represents the pathogenic allele. **(Middle Panel)** Cell-free DNA Composition and Fetal Genotypes: Maternal plasma cell-free DNA is a mixture of maternal (major fraction) and fetal (minor fraction) DNA. The diagram illustrates how the fetal genotype (AA, Aa, or aa) alters the total pool of alleles in circulation. Fetus AA (Homozygous Wildtype): Fetal contribution of “A” alleles dilutes the relative concentration of the “a” allele (A > a). Fetus Aa (Heterozygous Carrier): Fetal contribution mirrors the maternal background, maintaining allelic balance (A = a). Fetus aa (Homozygous Pathogenic): Fetal contribution of “a” alleles enriches the relative concentration of the “a” allele (A < a). **(Bottom Panel)** Quantitative RMD Analysis: The table demonstrates the theoretical calculation of the pathogenic allele fraction (a%) in maternal plasma, assuming a fetal fraction (FF) of 10%.Normal (AA): The absence of fetal “a” alleles results in a reduced total “a” fraction (45%). Carrier (Aa): The fraction remains at equilibrium (50%).Patient (aa): The contribution of fetal “a” alleles shifts the total fraction to 55%, which is detectable by sensitive molecular counting methods.

Despite its utility, RMD faces several significant limitations. RMD is inherently targeted to known mutation sites and therefore may miss variants of uncertain significance or novel pathogenic mutations, limiting its utility for rare-disease discovery and comprehensive genomic screening. Clinical translation is further constrained by a lack of standardized laboratory protocols, quality control frameworks, and large multicenter validation cohorts; many reports to date derive from small, heterogeneous series. To enable routine clinical adoption, future efforts should expand detectable variant spectra, streamline and automate analytical pipelines to reduce technical barriers, and establish consensus operational and quality standards validated in large prospective cohorts (47) (Figure 2).

### 3.2 Relative haplotype dosage (RHDO)

Relative haplotype dosage (RHDO) is an advanced high-throughput framework for non-invasive fetal haplotyping that leverages cell-free fetal DNA. RHDO combines dense SNP genotyping or sequencing with quantitative statistical inference—including sequential probability ratio tests, hidden Markov models, and Bayesian approaches—to detect subtle allelic imbalance at phased biallelic loci in maternal plasma and thereby infer fetal haplotypes with high confidence (48–52).

For autosomal recessive disorders, conventional RHDO workflows require parental genomic DNA and an affected proband to phase parental haplotypes; for X-linked conditions, maternal samples alone are sufficient to identify high-risk maternal haplotypes. Using a hidden Markov model–based haplotype inference pipeline, Chen et al. reported a 94.1% technical success rate and 99.1% verification accuracy among 4,356 thalassemia carriers (53). Further algorithmic refinements have improved sensitivity at low fetal fractions, with reported detection thresholds as low as 1% and an average threshold of approximately 6.2%, supporting earlier and more reliable prenatal diagnosis (40).

**Figure 3:**
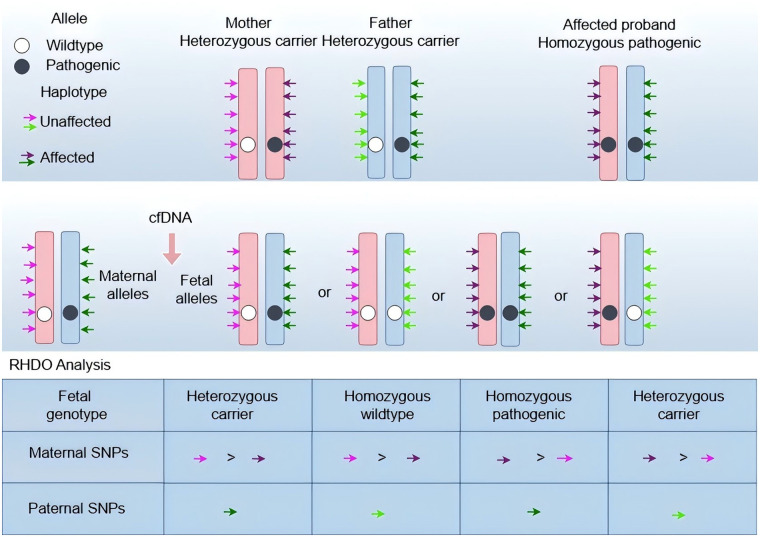
Principle of the relative single dose method. **(Top Panel)** Haplotype phasing: haplotype phasing is established using genomic DNA from the parents and an affected proband. Informative Single Nucleotide Polymorphisms (SNPs) flanking the locus of interest are identified to distinguish the parental haplotypes. (1) Maternal haplotypes: the wildtype allele (white circle) is linked to the “pink” SNP haplotype, while the pathogenic allele (dark circle) is linked to the “purple” SNP haplotype. (2) Paternal haplotypes: the wildtype allele is linked to the “light green” SNP haplotype, and the pathogenic allele is linked to the “dark green” SNP haplotype. (3) The affected proband (homozygous pathogenic) confirms the linkage phase of the high-risk alleles. **(Middle Panel)** Cell-free DNA Composition: In maternal plasma, cell-free DNA comprises a significant component of maternal DNA and a minor component of fetal DNA. The total pool of alleles reflects the maternal background superimposed by the fetal contribution, resulting in subtle allelic imbalances depending on the fetal genotype. **(Bottom Panel)** RHDO Analysis: Fetal genotype prediction is achieved by analyzing the allelic ratio of informative SNPs. (1) Maternal Inheritance (Dosage Analysis): The inheritance of the maternal allele is determined by measuring the dosage imbalance between the two maternal haplotypes. An overrepresentation (dosage >1) of the SNPs associated with a specific haplotype indicates its transmission to the fetus. For example, an excess of “purple” SNPs relative to “pink” SNPs ($Purple >Pink$) indicates the inheritance of the maternal pathogenic allele. (2) Paternal Inheritance (Direct Detection): the inheritance of the paternal allele is determined by the qualitative detection of paternal-specific SNPs (green arrows) that are absent in the maternal genome. The presence of “dark green” SNPs confirms the transmission of the paternal pathogenic allele. Diagnosis: The combination of maternal dosage analysis and paternal allele detection allows differentiation among heterozygous carriers, homozygous wildtype fetuses, and homozygous pathogenic fetuses.

A key limitation of conventional RHDO is its reliance on familial reference haplotypes—typically from an affected proband or parental samples—to phase parental alleles. Recent technological advances have reduced this dependency: linked read sequencing, targeted long-range amplification strategies, and third-generation long-read platforms enable haplotype reconstruction and non-invasive fetal phasing without requiring additional family members (54, 55) (Figure 3).

## 4 Clinical applications of non-invasive prenatal testing for monogenic diseases

### 4.1 Paternal inheritance and *de novo* mutations

Early clinical applications of NIPT employed polymerase chain reaction (PCR) on cell-free fetal DNA (cffDNA) to detect discrete fetal sequences. PCR-based assays reliably determined fetal sex via Y chromosome markers (e.g., SRY, DYS14) and assessed paternal inheritance of the RHD gene to guide management of hemolytic disease of the newborn (46).

As sequencing and analytical technologies have advanced, NIPT applications have expanded to include monogenic diseases. Current methods can detect *de novo* variants, identify paternally inherited alleles, and rule out paternal contributions in autosomal recessive cases where parents carry distinct mutations. However, PCR-based assays remain limited to interrogation of single, predefined mutations. They are therefore suboptimal for cases with complex phenotypes, multiple pathogenic variants, or when broad genomic interrogation is required to achieve a diagnosis.

Advances in next-generation sequencing (NGS) have substantially broadened the scope of NIPT for monogenic diseases. Targeted NGS panels enable simultaneous assessment of multiple variant types within a gene and interrogation of numerous disease-associated genes, permitting reliable detection of paternally inherited and *de novo* pathogenic single-nucleotide variants (SNVs) from cffDNA. Recent multicenter evaluations report high diagnostic performance for targeted NIPT—combined positive predictive values approaching 98.9%, sensitivities of 88.9%–100%, and specificities of 98.1%–100%—with a concomitant reduction in inconclusive results (56). Unbiased approaches (non-invasive fetal WES/WGS) further enable discovery of novel or unexpected pathogenic variants with comparable specificity and sensitivity for paternal and *de novo* events, though at greater analytical complexity and cost. Ongoing reductions in sequencing expense, expanding gene coverage, and accumulating multicenter clinical data—including extensive cohort studies in China—support translational momentum toward broader clinical implementation of non-invasive single gene testing.

### 4.2 Maternal inheritance and x-linked inheritance detection

NIPT of maternally inherited variants remains technically more challenging than the detection of paternally inherited or *de novo* mutations, mainly because of the abundance of maternal background cell-free DNA, which confounds allele quantification (23). Accurate inference, therefore, requires high-precision dosage analyses that quantify relative wild-type and mutant allele proportions in maternal plasma. Combined analytical frameworks—notably, relative haplotype dosage (RHDO), integrated with robust bioinformatics phasing and error-suppression algorithms—have demonstrated the ability to resolve fetal genotypes in autosomal recessive disorders by leveraging parental haplotypes and subtle allelic imbalance. Continued improvements in fetal fraction estimation, molecular barcoding, and statistical modeling are essential to increase the sensitivity and specificity of maternal allele detection for reliable clinical application.

In 2024, a Guangzhou group applied RHDO combined with targeted NGS for NIPT of phenylketonuria (PKU) in 61 pregnancies at 7–12 weeks' gestation, reporting a 96.7% technical success rate and 100% concordance in determining fetal inheritance of maternal PAH mutations (57). Concurrently, whole-genome–based multimodal pipelines that integrate fragmentomics, Bayesian inference, and machine-learning classifiers have improved the detection of *de novo* variants and the discrimination of parental SNVs, including maternally inherited homozygous biallelic events. Clinical validations indicate these integrated approaches increase the accuracy and breadth of non-invasive detection of pathogenic variants and trend toward enhanced sensitivity for maternal SNVs (23).

Despite technological progress, diagnostic sensitivity for fetal variants that share maternal alleles remains substantially lower than for paternally inherited or *de novo* mutations. Single cell sequencing—by isolating intact fetal cells and thereby eliminating the confounding maternal cell-free DNA background—offers clear technical advantages for resolving maternally inherited variants and compound heterozygosity. Emerging evidence supports single-cell approaches as a promising strategy to address current limitations in maternal allele detection and to improve the completeness and clinical reliability of non-invasive prenatal genomics (38).

## 5 Challenges of non-invasive monogenic disease diagnosis in clinical practice

### 5.1 Detection sensitivity and specificity

Despite substantial technological progress, NIPT of monogenic diseases continues to face persistent sensitivity and specificity limitations. Specific loci—such as FMR1, F8, and CYP21A2—remain refractory to routine cell-free DNA-based next-generation sequencing because fragmented cell-free DNA is ill-suited to detect exon- or subexon-level copy number changes and repeat expansions, thereby reducing diagnostic accuracy (47). Diagnostic performance also varies by inheritance mode: paternally inherited and *de novo* variants in autosomal dominant disorders are generally detected with high sensitivity and specificity (22), whereas autosomal recessive conditions pose greater challenges when the mother is a carrier, since abundant maternal mutant alleles in plasma can mask the fetal signal and degrade both sensitivity and specificity (30).

Fetal fraction (FF) is a critical determinant of assay performance; low FF (e.g., <10%, and notably <4%) markedly reduces the detectability of fetal variants and increases the risk of false negatives. Although quantitative dose-based methods can partially mitigate low FF effects, clinical practice often recommends repeat sampling at later gestational ages to increase FF and improve diagnostic confidence (8, 9).

### 5.2 Population applicability and detection efficacy

Current clinical validation of NIPT for monogenic diseases has been conducted predominantly in high-risk cohorts—pregnancies with fetal structural anomalies, a family history of pathogenic variants, or prior affected pregnancies—where higher pretest probability improves positive predictive value. Extrapolation to the general obstetric population is limited by selection bias and a lack of extensive, multicenter prospective data (47). Because monogenic disease prevalence is low, expanding screening to include ultra-rare or adult-onset conditions would increase coverage but complicate interpretation and raise the risk of false positives: even assays with high sensitivity and specificity yield lower PPVs when prior probability is low, potentially causing unnecessary maternal anxiety, additional invasive testing, and higher healthcare costs (13).

Before population-level deployment, rigorous real-world performance evaluations are required to define which conditions are appropriate for screening, to establish detection thresholds and reporting standards, and to develop consensus criteria for target populations. Equally important are structured genetic counseling pathways, standardized laboratory and reporting practices, and follow-up care frameworks to address the clinical, ethical, and socioeconomic implications of broader NIPT implementation.

### 5.3 Ethical, policy, and healthcare coverage challenges in clinical application

As NIPT for monogenic diseases moves toward broader clinical implementation, technical advances have exposed substantial ethical, policy, and coverage challenges. Genome-wide cell-free DNA assays (WES/WGS) frequently yield variants of uncertain significance or low penetrance alleles with variable expressivity, limiting clear clinical actionability and potentially increasing maternal anxiety and downstream interventions. Detection of apparently pathogenic variants (e.g., COL1A1) in phenotypically normal fetuses exemplifies the dilemma of overdiagnosis and raises difficult questions about the balance between potential benefit and harm.

Public understanding of monogenic disease, test limitations, and result uncertainty remains limited, underscoring the necessity for comprehensive pre- and post-test counseling to avoid coerced or misinformed decisions. Expanding NIPT from high-risk and assisted reproduction settings into routine prenatal care further blurs the boundary between screening and diagnosis, with attendant ethical implications for fetal interests and parental reproductive choices—particularly when pregnancy termination is considered based on nonconfirmatory screening results. Addressing these issues will require multidisciplinary consensus on clinical utility thresholds, standardized reporting and counseling frameworks, and policy measures governing test use and reimbursement.

The absence of standardized disease inclusion criteria has produced marked heterogeneity in which conditions are targeted for NIPT across institutions, undermining consistency in test composition and quality. Methodological validation is further compromised by a lack of unified performance standards—particularly for technically challenging variant classes such as exon-level copy number changes and repeat expansions—thereby limiting inter-laboratory comparability. Population-scale screening would also require extensive genetic counseling capacity to interpret results, convey disease natural history, and guide management; however, a global shortage of trained counselors impedes such deployment and raises concerns about informed consent and result misinterpretation (58). Counselors must carefully weigh the benefits of early intervention for actionable conditions against the harms of overdiagnosis and unnecessary interventions (59). Establishing consensus criteria for target conditions, standardized analytical and reporting frameworks, and scalable counseling models is therefore essential for safe, equitable, and clinically meaningful implementation of NIPT for monogenic disease.

Expanding genetic screening to include additional genes or rare disorders will increase per-test costs and, when applied at the population scale, could substantially burden healthcare systems. Although sequencing costs have declined, comprehensive cost–benefit and budget-impact analyses are required to evaluate the value of large-scale NIPT programs, because the low prevalence of many rare conditions may limit cost-effectiveness and complicate coverage decisions.

Currently, only a few countries have incorporated non-invasive monogenic screening into public insurance with defined scopes; for example, Japan began subsidizing single-gene tests for over 200 conditions in June 2024 (60). In most jurisdictions—including China—such testing remains predominantly out of pocket or selectively reimbursed for high-risk cases, exacerbating disparities in access by socioeconomic status and geography. Policy frameworks must therefore balance population health goals with individual autonomy, addressing whether and how screening for severe conditions should be prioritized, regulated, and equitably financed.

## 6 Conclusion

Non-invasive prenatal testing (NIPT) for monogenic diseases has progressed substantially. Targeted sequencing yields high accuracy for known pathogenic loci and reliably detects paternally inherited and *de novo* variants. Genome-wide approaches (WES/WGS) extend detection to a broader spectrum of *de novo* and unexpected variants. At the same time, single-cell sequencing—still largely investigational—offers a route to eliminate maternal cell-free DNA interference. Quantitative analytical frameworks such as RMD and RHDO further improve genotype inference from mixed maternal–fetal samples.

Despite these advances, essential limitations remain. Accurate detection of maternally inherited variants is constrained by abundant maternal DNA; low fetal fraction reduces assay robustness; many methods lack validation across diverse populations; and ethical issues, variable standardization, limited genetic counseling capacity, and inconsistent reimbursement hinder practical deployment. Continued technical refinement, large-scale multiethnic validation, consensus standards, and integrated counseling and policy frameworks will be essential to realize the full clinical potential of NIPT for monogenic disease prevention and management.
